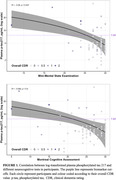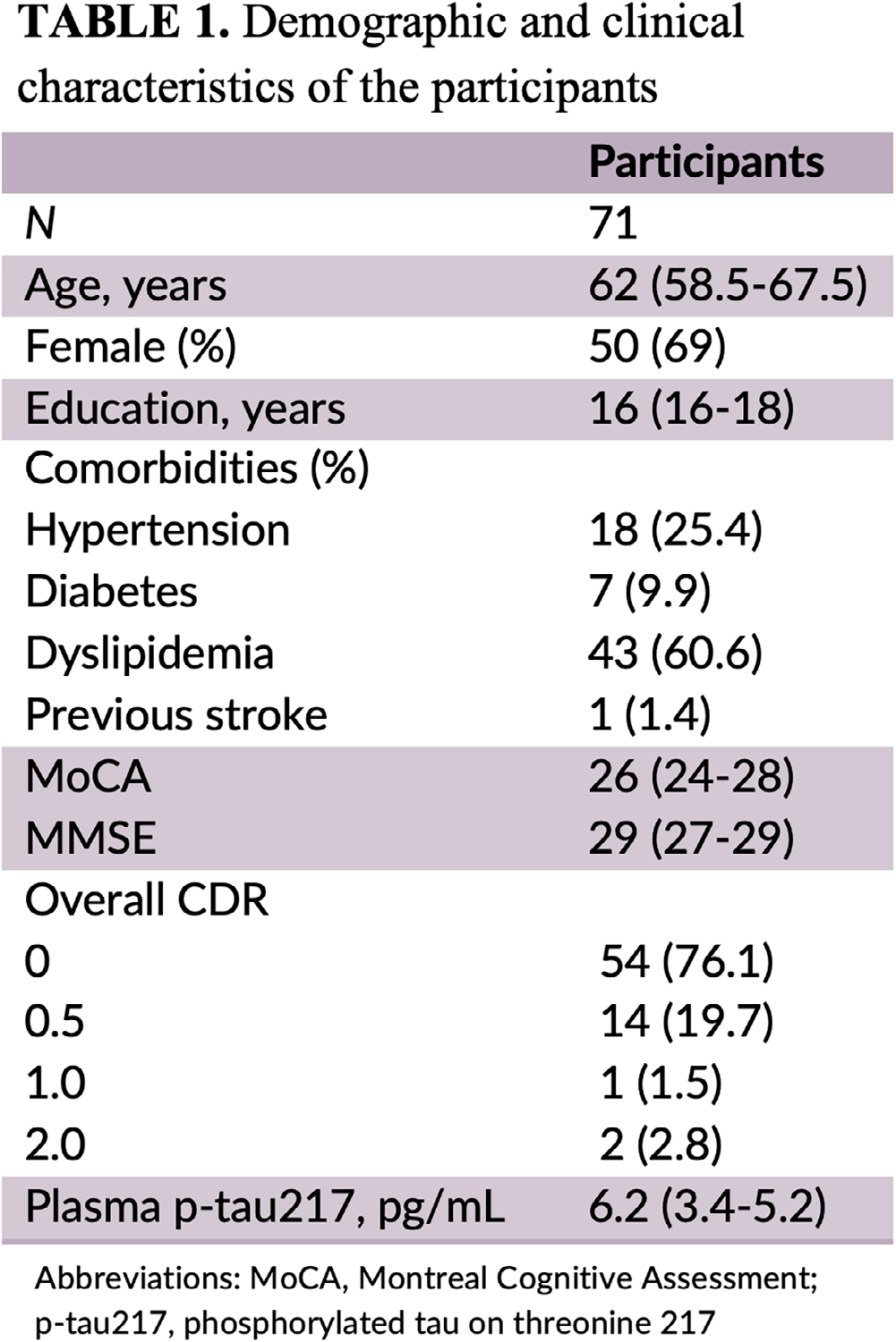# Plasma phosphorylated tau 217 in relation to Alzheimer’s prevalence and cognition in Thailand

**DOI:** 10.1002/alz.090601

**Published:** 2025-01-09

**Authors:** Pasin Hemachudha, Poosanu Thanapornsangsuth, Watayuth Luechaipanit, Thiravat Hemachudha

**Affiliations:** ^1^ Thai Red Cross Emerging Infectious Diseases Health Science Centre, King Chulalongkorn Memorial Hospital, Bangkok Thailand; ^2^ Faculty of Medicine, Chulalongkorn University, Bangkok Thailand; ^3^ Elderly Health Care Center, Queen Savang Vadhana Memorial Hospital, Sriracha, Chonburi Thailand; ^4^ Chula Neuroscience Center, King Chulalongkorn Memorial Hospital, Bangkok Thailand; ^5^ Division of Neurology, Department of Medicine, Faculty of Medicine, Chulalongkorn University, Bangkok Thailand; ^6^ King Chulalongkorn Memorial Hospital, Bangkok Thailand

## Abstract

**Background:**

Biomarkers for Alzheimer's disease (AD) in blood samples has the potential to facilitate early diagnosis and improve the accuracy of AD diagnosis. Plasma phosphorylated tau (p‐tau) is a key biomarker for AD; however, its utility for estimating the prevalence of AD and screening for cognitive impairment is still limited.

**Method:**

The study recruited 71 participants over the age of 40. Plasma p‐tau 217 levels and neuropsychological data were measured. AD prevalence was estimated using a pre‐defined cut‐off derived from an independent cohort with biomarker‐defined AD status according to the current framework (Jack et. al., 2018). The correlation between plasma p‐tau 217 levels and neuropsychological data were also evaluated.

**Result:**

The median age of participants was 62 years and overall clinical dementia rating of 0. The median Montreal cognitive assessment (MoCA) and Mini‐Mental State Examination (MMSE) score was 26 and 29 consecutively. Plasma p‐tau 217 levels showed a negative correlation with both neurocognitive tests (MoCA, Rho = ‐0.082, P = 0.5; MMSE, Rho = ‐0.26, P = 0.027). The estimated prevalence of AD was 16.9% (95% CI 9.9‐27.3) when using plasma p‐tau 217 as a biomarker, which was lower than the prevalence estimated using amyloid abnormalities of 30.1% (Jansen et. al., 2022).

**Conclusion:**

The results of this study suggest the possibility of plasma p‐tau 217 for estimating AD prevalence and cognition. It may represent those at risk of clinical onset. However, further studies are needed to investigate its utility in risk stratification, and cognitive performance assessment.